# Stacked survival models for residual lifetime data

**DOI:** 10.1186/s12874-021-01496-3

**Published:** 2022-01-07

**Authors:** James H. McVittie, David B. Wolfson, Vittorio Addona, Zhaoheng Li

**Affiliations:** 1 Department of Mathematics and Statistics, McGill University, Montreal, Canada; 2 Department of Mathematics, Statistics and Computer Science, Macalester College, St.Paul, USA

**Keywords:** Survival analysis, Residual lifetime data, Nonparametric estimation, Stacking

## Abstract

When modelling the survival distribution of a disease for which the symptomatic progression of the associated condition is insidious, it is not always clear how to measure the failure/censoring times from some true date of disease onset. In a prevalent cohort study with follow-up, one approach for removing any potential influence from the uncertainty in the measurement of the true onset dates is through the utilization of only the residual lifetimes. As the residual lifetimes are measured from a well-defined screening date (prevalence day) to failure/censoring, these observed time durations are essentially error free. Using residual lifetime data, the nonparametric maximum likelihood estimator (NPMLE) may be used to estimate the underlying survival function. However, the resulting estimator can yield exceptionally wide confidence intervals. Alternatively, while parametric maximum likelihood estimation can yield narrower confidence intervals, it may not be robust to model misspecification. Using only right-censored residual lifetime data, we propose a stacking procedure to overcome the non-robustness of model misspecification; our proposed estimator comprises a linear combination of individual nonparametric/parametric survival function estimators, with optimal stacking weights obtained by minimizing a Brier Score loss function.

**Supplementary Information:**

The online version contains supplementary material available at (10.1186/s12874-021-01496-3).

## Introduction

The Canadian Study of Health and Aging (CSHA) was a nation-wide study whose primary goal was to determine the prevalence of dementia in five different regions in Canada [1, 2]. In 1991, at the first stage of the study (CSHA-1), approximately 10,000 individuals over the age of 65 were screened for various types of dementia. A total of 823 participants were classified at CSHA-1 as having either probable Alzheimer’s disease, possible Alzheimer’s disease or vascular dementia. They were followed for a subsequent five years until the second stage of the study in 1996 (CSHA-2). Death dates were recorded for those who died between 1991 and 1996 together with the censoring dates of those who were lost to follow-up or survived until 1996. The onset dates of the participants who screened positive at CSHA-1 were retrospectively reported through the recollections of their caregivers. The observed (right-censored) survival times were the durations of time from reported onset to failure/censoring. The resulting failure times were therefore considered to be left-truncated and right-censored as typically occurs in a prevalent cohort study with follow-up [3]. Suppose, further, it is assumed that the underlying process that defines all of the onset dates, including those not associated with the observed prevalent cohort, is a stationary Poisson process. Then we shall say that our inference is carried out “under the stationarity assumption” [4], an assumption that is crucial for the methods proposed in this article.

Now, due to the onset date recording protocols in the CSHA as well as the insidious symptomatic onset of dementia, the true failure/censoring times were almost surely measured with error. Under the stationarity assumption, in more general prevalent cohort studies with follow-up, uncertainty in the onset dates can be accounted for in at least two ways. First, under the assumption that the failure time distribution is defined parametrically, McVittie et al. defined an adjusted classical measurement error model for the reported onset dates, to derive maximum likelihood estimators for the unknown failure time distribution parameters [5].

A second approach, under stationarity, is to discard the information contained in the uncertain onset times by using only the residual lifetimes for estimation of the survival distribution. The residual lifetimes extend from the date of screening to the date of failure/censoring. As the residual lifetimes are not dependent on the onset dates, these durations are error free. It should be noted that without stationarity it is impossible to make inference about the failure time distribution based only on observation of the residual lifetimes. In order to review the literature on this approach, we use notation which will be more systematically introduced in Section 2. Let *S*_*U*_(·) be the underlying (unbiased) survival function (the estimation target) and let *μ* be its mean. Let *f*_res_(·) be the residual lifetime probability density function (pdf). Then, this setting can be regarded as equivalent to the scenario in which the residual lifetimes (with pdf *f*_res_(·)) of a stationary renewal process, with interarrival time survivor function *S*_*U*_(·), are the observations [6]. Exploiting this equivalence, and a well known property of stationary renewal processes [7–9], it can be shown that,

1 $$ \begin{aligned} f_{\text{res}}(\cdot) &= S_{U}(\cdot)/\mu \\ &= S_{U}(\cdot) f_{\text{res}}(0)  \end{aligned}  $$

**Fig. 1:**
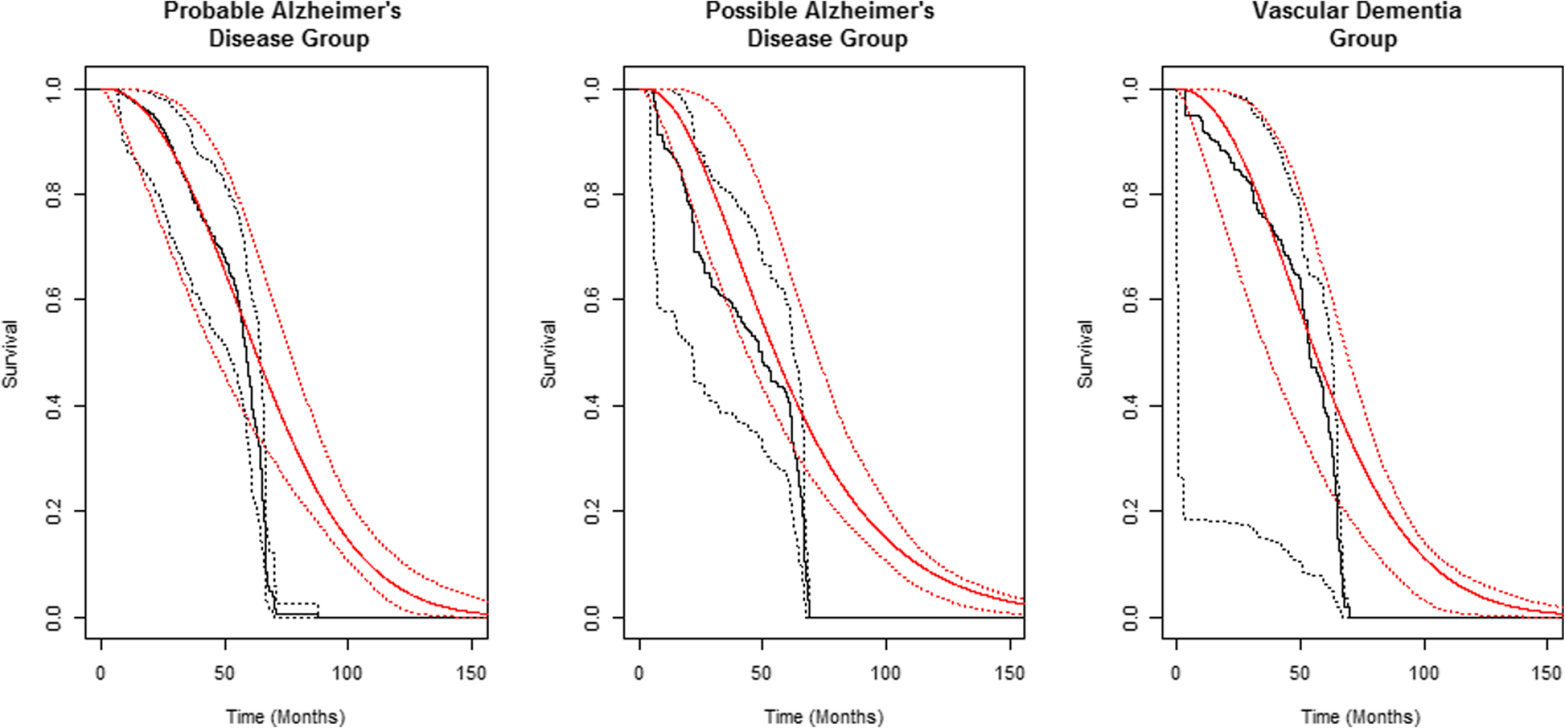
Corrected non-parametric maximum likelihood survival function estimates with 95% bootstrapped pointwise confidence limits (black) for varying dementia subgroups in the Canadian Study of Health and Aging along with stacked survival function estimates with 95% bootstrapped pointwise confidence limits (red)

It follows from (1), that if *S*_*U*_ is parametrically defined, its maximum likelihood estimator (MLE) can be found by finding the MLEs of the parameters that define *f*_res_; the (possibly censored) residual lifetimes can be used to construct the likelihood function [7, 8]. Non-parametric estimation of *S*_*U*_, however, requires much more care and has been the subject of much research both in the context of renewal processes and survival analysis. The difficulty arises from the instability in $\hat {S}_{U}(\cdot) = \hat {f}_{\text {res}}(\cdot)/\hat {f}_{\text {res}}(0)$, due to its dependence on the boundary-point estimator $\hat {f}_{\text {res}}(0)$. For uncensored data, and recognizing that by (1), *f*_res_(*u*) is non-increasing in *u*, Grenander showed that the NPMLE of *F*_*U*_(·)=1−*S*_*U*_(·), is the least concave majorant of the empirical distribution function [10]. Woodroofe and Sun proposed a penalized maximum likelihood procedure to consistently estimate the residual lifetime density function at the boundary [11]. For right-censored residual lifetime data, the least concave majorant of the cumulative distribution function estimated using the Kaplan-Meier estimator, in place of the empirical survival function, is no longer the NPMLE [12, 13]. Denby and Vardi proposed an iterative EM based algorithm to determine the NPMLE using right-censored residual lifetime data [14]. They also proposed a “corrected” NPMLE to account for the bad behavior of the NPMLE at times close to zero. Huang and Zhang remark that in the right-censored setting, the boundary point estimator using the NPMLE is both unstable and inconsistent [15]. Although an approach combining the Denby and Vardi algorithm with the penalization procedure of Woodroofe and Sun has been alluded to in the literature, it has not been formally described and compared to other methodologies. Recently, Westling and Carone surveyed the asymptotic properties of nonparametric survival function estimators subject to monotonicity constraints [9]. Using current duration data, Keiding studied the behaviour of the corrected NPMLE and associated parametric models for modelling the time to pregnancy [7, 8]. He remarked that the confidence intervals obtained from the corrected NPMLE were wide due to the unstable boundary estimation problem at time *t*=0 [7, 8]. Using data collected from the CSHA, this phenomenon is most evident in the nonparametric maximum likelihood survival function estimates for subjects with possible Alzheimer’s disease and vascular dementia (see Fig. 1).

There is another disadvantageous feature of the NPMLE of *S*_*U*_: If the study follow-up period is short, the NPMLE is unlikely to estimate *S*_*U*_ well, beyond the largest observation in the sample. This feature is demonstrated in the survival curve estimates of Fig. 1 as all three curves drop to near 0 at approximately 60 months. When we started on this research we anticipated using an estimator, based on the (possibly censored) residual lifetimes, that includes both the NPMLE and several suitable parametric estimators, thereby counteracting the drawbacks of each of these two types of estimator. We reasoned that in the context of standard right-censored failure time data, Wey et al. had proposed a stacking procedure which successfully combines non/semi-parametric and parametric survival function estimators into a single estimator of the underlying survival distribution [16, 17]. To our surprise, however, particularly when applied to right-censored residual lifetime data with short follow-up, we found that there was little advantage to including the NPMLE in the stacking procedure; that is, the NPMLE received very little weight.

We adapt the stacking approach of Wey et al. to estimate *S*_*U*_ using right-censored residual lifetime data. Our goals are to: (i) enable estimation of the survival function past the last observed failure/censoring time when follow-up is short, (ii) provide an estimation procedure which is robust to model misspecification and (iii) reduce the width of the confidence intervals that would be obtained from the NPMLE alone. In Section 2, we introduce notation for prevalent cohort studies with follow-up and specify how the procedure of Wey et al. is modified for residual lifetime data. In Section 3, we use simulated failure time data to examine the performance of the stacked estimator against estimators based on individual models. We apply our stacking methodology to the CSHA data set in Section 4 and provide some concluding remarks in Section 5.

## Notation and methodology

**Fig. 2:**
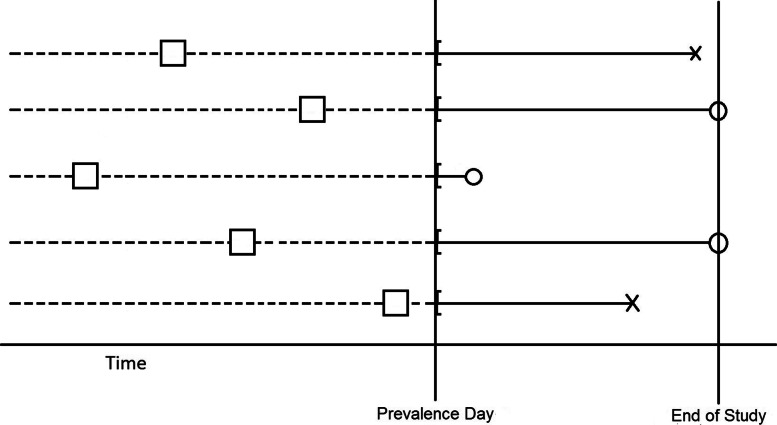
A depiction of a sample of right-censored residual lifetime data. The open circles represent the calendar dates of censoring and the crosses represent the calendar dates of failure. Brackets represent the earliest confirmed time for the start of the failure/censoring time durations. Dashed lines represent uncertainty in the measurement of the underlying onset dates (represented by open squares)

Let (*O,T*) denote the random variable pair consisting, respectively, of a generic onset date drawn from a stationary Poisson process and a generic failure time with survival function *S*_*U*_(·) where *O* is independent of *T*. Let the fixed constant *R* denote the screening date at which the prevalent cohort is determined and which we define as “prevalence day”. The prevalent cohort then consists of subjects with (onset, failure time) pairs such that *O*<*R* and *O*+*T*>*R*. Let *C* denote the censoring time (measured from prevalence day) with cumulative distribution *G*(·) corresponding to subjects who are either lost to follow-up or have not failed by the end of the study (administratively censored). The full prevalent cohort data then comprises the triples $\{(A_{i}, Y_{i}, \delta _{i}) = (R - O_{i}, \min (T_{i}, R-O_{i}+C_{i}), 1_{\{ T_{i} < R-O_{i}+C_{i}\}}): T_{i} > R - O_{i}, i = 1, 2,..., n \}$. As the residual lifetimes consist only of the failure/censoring times measured from prevalence day and their associated indicator functions, they are given by the pairs $\{(V_{i}, \delta _{i}) = (\min (T_{i} - (R - O_{i}), C_{i}), 1_{\{T_{i} < R-O_{i}+C_{i}\}}): T_{i} > R - O_{i}, i = 1, 2,..., n\}$. For a depiction of residual lifetime data, see Fig. 2.

For convenience, we repeat Eq. 1, now numbered (2):

2 $$ f_{\text{res}}(\cdot) = \frac{S_{U}(\cdot)}{\mu}   $$

where $\mu = \mathbb {E}(T)$. By evaluating the pdf *f*_res_, at time *t*=0, and utilizing the property that *S*_*U*_(0)=1, from Eq. 2, it follows that $\mu = \frac {1}{f_{\text {res}}(0)}$, and hence *f*_res_(·)=*S*_*U*_(·)*f*_res_(0). This suggests

3 $$ \hat{S}_{U}(t) = \frac{\hat{f}_{\text{res}}(t)}{\hat{f}_{\text{res}}(0)}   $$

as a plug-in estimator for *S*_*U*_. When *S*_*U*_(·;***θ***) is defined parametrically, for some unknown p-dimensional parameter ***θ***, the likelihood function is given by [7]:

4 $$ \mathcal{L}(\boldsymbol{\theta}) = \prod_{i=1}^{n} \left(\frac{S_{U}(v_{i}; \boldsymbol{\theta})}{\mu(\boldsymbol{\theta})} \right)^{\delta_{i}} \left(\int_{v_{i}}^{\infty} \frac{S_{U}(x; \boldsymbol{\theta})}{\mu(\boldsymbol{\theta})} dx \right)^{1-\delta_{i}}   $$

Let $\hat {\boldsymbol {\theta }}$ be the MLE of ***θ***, obtained from (4). Although the parametric maximum likelihood estimator $S_{U}(\cdot ; \hat {\boldsymbol {\theta }})$ is model-dependent and possibly biased, it has a smaller variance than does its non-parametric counterpart in (3).

One approach which combines nonparametric and parametric estimators is through the machine learning procedure known as *stacking*. A stacked survival function estimator is a weighted linear combination of sub-model survival function estimators for which the optimal weights are determined through optimization of a particular loss function. Here, we consider the approach of Wey et al. which allows for right-censoring of the data [16]. We begin by proposing *m*−1 parametric models for *f*_res_(·): *f*_res,1_(·;***θ***_1_),*f*_res,2_(·;***θ***_2_),...,*f*_res,*m*−1_(·;***θ***_*m*−1_) for *m*≥2. Let $\hat {f}_{\text {res},1}(\cdot), \hat {f}_{\text {res},2}(\cdot),..., \hat {f}_{\text {res},m}(\cdot) = f_{\text {res},1}(\cdot ; \hat {\boldsymbol {\theta }}_{1}), f_{\text {res},2}(\cdot ; \hat {\boldsymbol {\theta }}_{2}),...., f_{\text {res},m-1}(\cdot ; \hat {\boldsymbol {\theta }}_{m-1}), \hat {f}_{\text {res},m}(\cdot)$ be *m* estimators of *f*_res_(·), where $\hat {f}_{\text {res},m}(\cdot)$ is the (non-increasing) corrected NPMLE defined by Denby and Vardi, and $\hat {f}_{\text {res}, i}(\cdot)$ is the parametrically defined MLE for *i*=1,2,...,*m*−1 [14]. We define a stacked density estimator of *f*_res_(·) as:

$${}\hat{f}_{\text{res}, \text{stack}}(\cdot) = \alpha_{1} \hat{f}_{\text{res},1}(\cdot) + \alpha_{2} \hat{f}_{\text{res},2}(\cdot) +... + \alpha_{m} \hat{f}_{\text{res},m}(\cdot),$$

where *α*_*i*_∈[0,1] and $\sum _{i=1}^{m} \alpha _{i} = 1$. Since each $\hat {f}_{\text {res},i}(\cdot)$ is non-increasing, $\hat {f}_{\text {res},\text {stack}}$ is also non-increasing. By integrating the linear combination of pdf estimators, we obtain a stacked residual lifetime survival function estimator given by:

5 $$ {}\hat{S}_{\text{res}, \text{stack}}(\cdot) = \alpha_{1} \hat{S}_{\text{res},1}(\cdot) + \alpha_{2} \hat{S}_{\text{res},2}(\cdot) +... + \alpha_{m} \hat{S}_{\text{res},m}(\cdot)   $$

The general idea is to find the optimal weights $\hat {\alpha }_{1}, \hat {\alpha }_{2},..., \hat {\alpha }_{m}$ by minimizing an objective function of $\hat {S}_{\text {res},\text {stack}}(\cdot)$. Specifically, let $V^{\prime }_{i}(t) = \min (V_{i}, t), \delta ^{\prime }_{i}(t) = 1_{\{(\min (V_{i}, t) < C_{i})\}}, Z_{i}(t) = 1_{\{V_{i} > t\}}$ and let $\hat {G}(\cdot)$ be the Kaplan-Meier estimator of the residual censoring time distribution function. Following Wey et al., we minimize the inversely weighted (by the probability of censoring) objective function, the Brier score, to determine the optimal weights, $\hat {\alpha }_{1}, \hat {\alpha }_{2}$,..., $\hat {\alpha }_{m}$ [16]. To control for possible overfit- ting, we use cross-validation and evaluate the Brier score over a set of *s* specified evaluation points to obtain:

6 $$ \begin{aligned} \hat{\boldsymbol{\alpha}} &= \underset{\boldsymbol{\alpha}: \alpha_{k} \in [0,1]}{\arg\min} \sum_{r=1}^{s} \sum_{i=1}^{n} \frac{\delta_{i}'(t_{r})}{\hat{G}(Z_{i}(V'_{i}(t_{r})))}\\ & \quad\times \left\{ Z_{i}(t_{r}) - \sum_{k=1}^{m} \alpha_{k} \hat{S}_{\text{res}, k}^{(-i)}(t_{r}) \right\}^{2} \end{aligned}  $$

where the superscipt (−*i*) denotes that the estimate was determined by leaving the i^th^ observation out during the estimation procedure. Due to computational constraints, we performed 5 fold cross-validation and evaluated the optimal weight parameters over nine equally spaced out points covering the support of the observed residual lifetimes, as suggested by Wey et al. [16]. Finally, exploiting Eq. 6 to obtain $\hat {\boldsymbol {\alpha }}$, and Eq. 3 to obtain $\hat {S}_{U,m}(\cdot)$ from $\hat {f}_{\text {res},m}(\cdot)$, we define the stacked survival function estimator

7 $$ {}\hat{S}_{U, \text{stack}}(\cdot) = \hat{\alpha}_{1} \hat{S}_{U, 1}(\cdot) + \hat{\alpha}_{2} \hat{S}_{U, 2}(\cdot) +... + \hat{\alpha}_{m} \hat{S}_{U, m}(\cdot).   $$

The estimator presented in (7) is, admittedly, an ad-hoc proposal, but one that is necessary given our lack of access to data arising from the underlying survival distribution, but rather from the residual lifetime distribution.

## Simulations

Using (potentially) right-censored simulated residual lifetime data, we evaluated the stacking estimator. We examined the performance of the individual parametric/nonparametric estimators relative to the stacked estimator when the residual lifetime data were subject to random right-censoring as well as increasing proportions of administrative right-censoring. The goal was to assess the increasing advantage, as follow-up decreases, of using a stacking estimator with both the corrected NPMLE and parametric survivor functions in the stack. A general description of the simulations examined in this manuscript is given in Table 1.

**Table 1:** A summary of the simulation studies examining the performance of the proposed stacked survival model estimation procedure

Simulation Number	Simulation Study Description
Simulation 1	Weibull distributed failure times with various amounts of administrative censoring (10%, 20%, 30%, 40%) acting on the residual failure time data.
	Two stacked models fitted: All submodels, All submodels except Weibull
Simulation 2	Mixture model distributed failure times with 30% random censoring.
	One stacked model fitted: All submodels

To simulate a set of right-censored residual lifetime data, we first generated an onset date, *O*, from a Uniform distribution with support (0,50). We generated a failure time, *T*, from either a Weibull distribution with shape and scale parameters equal to 2 and 2, respectively or from a mixture model of Weibull, Log-Logistic, Log-Normal and Gamma distributions. The latter failure time distribution was used to assess the predictive performance of the stacked estimator when the underlying failure time distribution was not included in any of the parametric models included in the stack. With the addition of covariates, the methods described by Bender et al. may be used to simulate failure times from a proportional hazards model [18]. However, with our proposed methodology, we do not consider the inclusion of covariate data through a regression-type model. We sampled onset, failure time pairs (*O,T*), for which *T*>50−*O*, until a sample of size *n* was selected. The sampled residual failure times, *T*_*i*_−(50−*O*_*i*_) for *i*=1,2,...,*n* were then right-censored either by a constant *C*^∗^ to correspond to administrative censoring or by the random variable *C*_*i*_ drawn from an Exponential distribution to allow for random censoring (i.e. loss to follow-up).

**Fig. 3:**
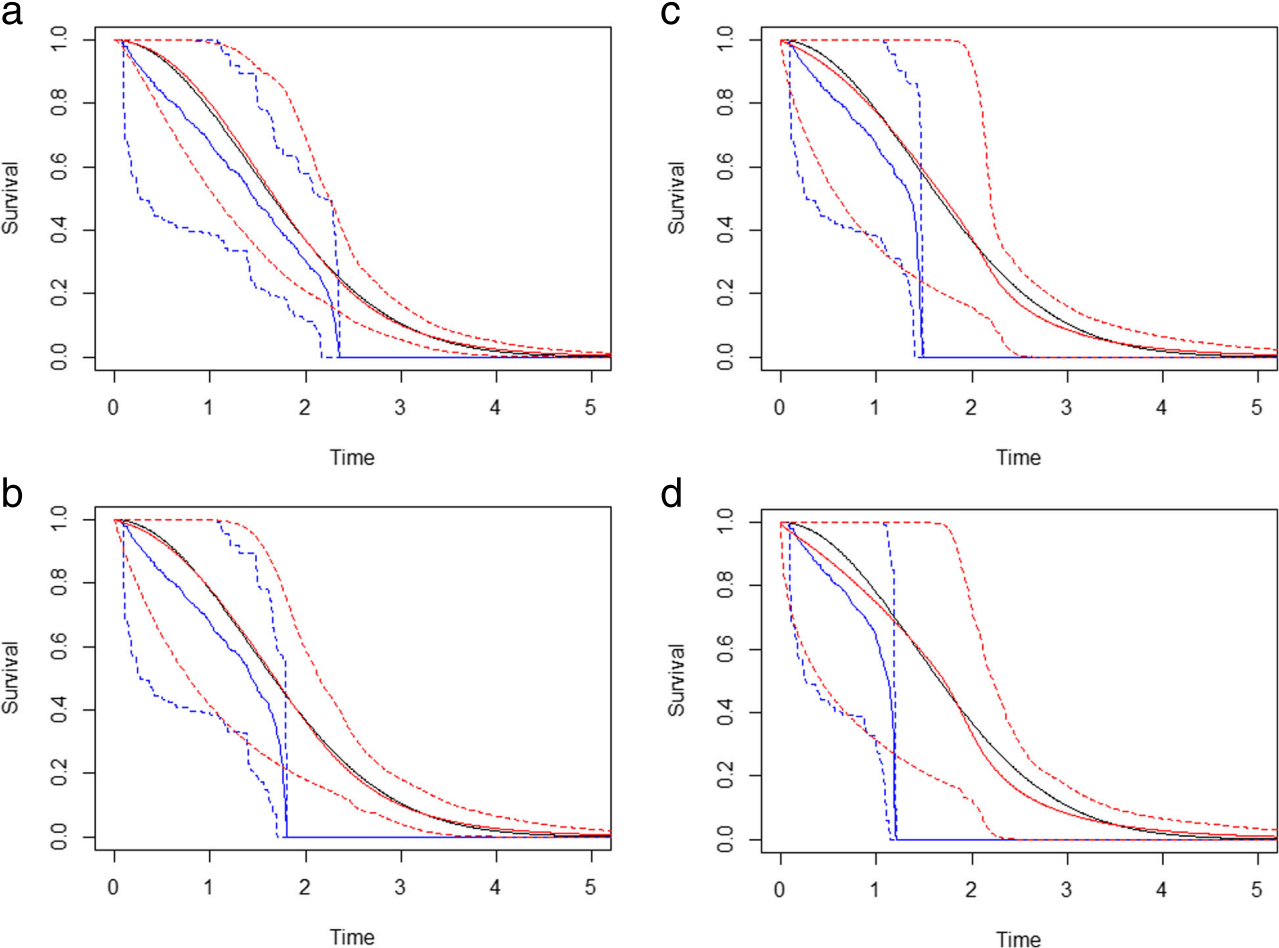
Graphical comparison of the NPMLE estimate (blue lines), stacked model estimate (without the Weibull submodel included) (red lines) relative to the underlying Weibull failure time survival function (black line) using samples of size 125 over 100 simulation runs with varying amounts of administrative censoring

In our first set of simulations, we assumed the underlying failure times were distributed according to a Weibull distribution and the residual failure times were administratively censored by moving up end-of-study dates to result in, respectively, 10%, 20%, 30% or 40% censoring. For each censoring percentage, we fit the corrected NPMLE, Weibull, Log-Logistic, Log-Normal and Gamma models. Using all five submodels, we determined the optimal stacking weights and computed the discrete integrated squared survival errors (DISSE) for the models when fitted separately and when combined in a stacked model. The DISSE is given by:

$$\text{DISSE} = \sum_{j=1}^{k} (t_{j} - t_{j-1}) (\hat{S}(t_{j}) - S_{0}(t_{j}))^{2}$$

where we defined a uniform mesh, 0=*t*_1_<*t*_2_<...<*t*_*k*_=50, to evaluate the predictive performance of the estimated survival functions over the support of the underlying survival function. The DISSE is the discretized version of the integral given by: $\int _{0}^{\infty } \left (\hat {S}(t) - S(t)\right)^{2} dt$. To evaluate this integral numerically, we proposed a uniform mesh over the majority of the support of the estimated/true survival functions ($\hat {S}$, *S*, respectively). The upper bound of the support was set to “50” as the underlying survival functions in both the Weibull simulations and mixture model simulations had negligible probability beyond this point. The gauge of the mesh was set to 0.1 but could be made finer for a better approximation to the integral. We also considered a second stacked model which included the corrected NPMLE and all parametric models except the Weibull (i.e., the true data generating model). We utilized samples of size 125 (i.e. 125 observed residual lifetimes) over 100 simulation runs and report the average DISSEs in Table 2 as well as the average weights for the stacked models in Table 3. The average survival function estimates and 95% pointwise confidence intervals using the NPMLE or stacked model (without the Weibull submodel) are plotted in Fig. 3. As the proportion of administrative censoring increases, we see that although the average DISSEs of the individual and stacked models all increase, the NPMLE DISSE increases at a much faster rate than those of the individual parametric models and the stacked models. This is expected as administrative censoring shortens the follow-up period and the range of the observed residual lifetime data thus affecting the nonparametric maximum likelihood estimator most severely. In Fig. 3, the corrected NPMLE is clearly biased beyond the administrative censoring times with narrow confidence interval widths in this range. Using the stacked model, we find that almost all of the weight is shifted away from the corrected NPMLE and to the correct underlying Weibull failure time distribution. When the Weibull model is excluded from the stack, the majority of the weight shifted to the Gamma model and the stacked model still yielded a smaller DISSE than the corrected NPMLE. The stacked model without the Weibull submodel appears roughly unbiased and exhibits narrower pointwise confidence intervals than the NPMLE when the censoring proportion is low.

**Table 2:** Average discrete integrated squared survival errors for individual and stacked models for a Weibull (2,2) failure time distribution with varying amounts of administrative censoring for samples of size 125 over 100 simulation runs

	Proportion of Administrative Censoring			
Model	10%	20%	30%	40%
NPMLE	0.09594	0.1639	0.2414	0.3645
Weibull	0.02776	0.03843	0.06635	0.09601
Log-Logistic	0.03244	0.03350	0.05696	0.08661
Log-Normal	0.03009	0.03660	0.06222	0.07615
Gamma	0.03056	0.04181	0.06025	0.08460
Stacked Model (all)	0.02877	0.04203	0.06680	0.09865
Stacked Model (w/o Weibull)	0.03049	0.04303	0.06546	0.09010

**Table 3:** Mean weights for a stacked model including all submodels or including all submodels except Weibull. The failure time data were generated according to a Weibull (2, 2) distribution with varying amounts of administrative censoring for samples of size 125 over 100 simulation runs

	Individual submodel type of stacked estimator				
Administrative Censoring Proportion	NPMLE	Weibull	Log-Logistic	Log-Normal	Gamma
10%	0.01394	0.08208	0.02781	0.04167	0.09579
	0.01607	N/A	0.05645	0.08028	0.8472
20%	5.586×10^−9^	0.9061	0.01439	0.04962	0.02992
	8.419×10^−9^	N/A	0.04088	0.08901	0.8701
30%	3.900×10^−9^	0.8503	0.03581	0.04999	0.06389
	5.520×10^−9^	N/A	0.07797	0.08018	0.8418
40%	2.169×10^−9^	0.7888	0.02656	0.01465	0.1700
	3.463×10^−9^	N/A	0.1319	0.04835	0.8197

**Fig. 4:**
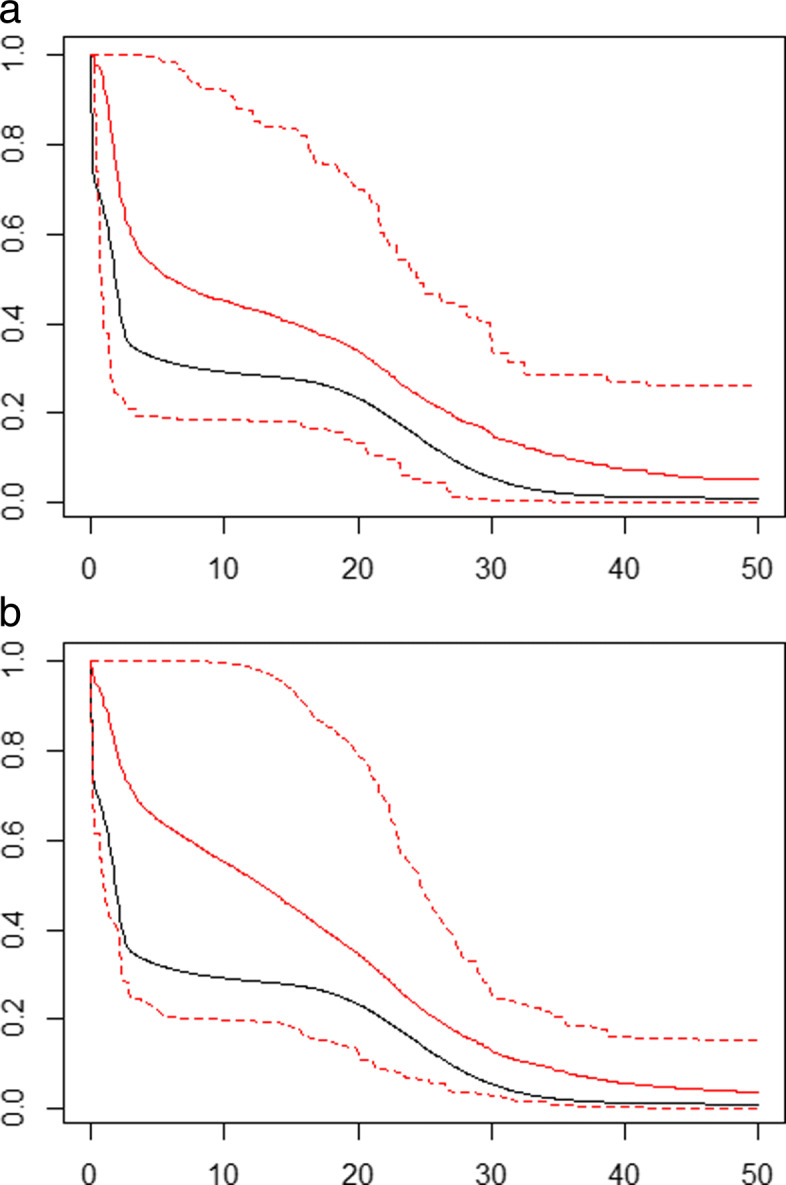
Graphical comparison of the individual model mean survival estimates (solid red line) with bootstrapped 95% pointwise confidence intervals (dotted red lines) relative to the underlying mixture failure time survival function (solid black line) using samples of size 125 over 100 simulation runs with 30% random censoring (Panel a - NPMLE, Panel b - Stacked Estimator)

Our second simulation considered residual failure time data that were generated from a mixture model. The failure time mixture model was comprised of four evenly weighted (25%) models consisting of the Weibull (shape, scale equal to 4, 2), Log-Logistic (shape, scale equal to 1, 2), Log-Normal (meanlog, standard deviation-log equal to -5 and 1) and Gamma (shape, scale equal to 25, 1) distributions. To generate a sampled failure time from the mixture model, first we made a single draw from a multinomial distribution with four states with equal probabilities of 0.25. The multinomial draw determined from which parametric model we sampled the failure time. Once the failure time was sampled, we sampled an onset time and then repeated the same left-truncating/right-censoring procedure as was conducted in the first set of simulations to generate residual lifetime data drawn from a prevalent cohort study with follow-up. We chose a mixture model in order to produce a survival function, with "kinks", that does not resemble the survival function of any of the standard parametric models used in survival analysis. With this mixture model, we anticipated that because of its flexibility, the NPMLE would out-perform any of the estimators based on the standard models, even the stacking model. We believe, though, that in most applications the survival function is unlikely to arise from a mixture. The residual failure times were randomly right-censored to allow for approximately 30% censoring. In this simulation scenario, there was no administrative censoring. We chose not to allow for administrative censoring in order to isolate the effect on the predictive performance of the stacked model when the underlying failure time model was not a member of the class of submodels included in the stacking procedure. We fit all five submodels individually and combined them in a stacked model. In Fig. 4, we plot the underlying mixture survival function in black with the corrected NPMLE and stacked survival function estimates, with their respectively bootstrapped 95% pointwise confidence intervals, in red. From the various plots, we find that the NPMLE tends to capture the general shape of the underlying survival function and captures the survival curve within its 95% pointwise confidence intervals. Other than the Gamma distribution, the individual parametric models do not capture the shape of the underlying survival function and for various models, their 95% confidence intervals do not capture the underlying survival curve at certain time points. Over 100 simulation runs, using the stacked model estimates, the mean weight for the corrected NPMLE was 0.4372 whereas the average weights for the other parametric models were 0.1475 (Weibull), 0.2581 (Log-Logistic), 0.01849 (Log-Normal) and 0.1387 (Gamma). The average DISSEs are listed in Table 4. Although the stacked model estimate did not perform as well as the corrected NPMLE with respect to the average DISSE, the stacked estimator yielded an improvement over the other parametric models. In addition, unlike the parametric estimators, there were no time points at which the stacked model estimator’s bootstrapped 95% pointwise confidence interval did not capture the underlying survival function.

**Table 4:** Average discrete integrated squared survival errors (DISSE) for individual and stacked models for a mixture failure time distribution with 30% random censoring for samples of size 125 over 100 simulation runs

Model	Average DISSE
NPMLE	1.478
Weibull	2.755
Log-Logistic	5.612
Log-Normal	4.655
Gamma	3.075
Stacked Model (all)	2.273

## Application

We demonstrated our proposed stacking estimator by using it to estimate survival with dementia from forward recurrence time data obtained from the CSHA, as described at the beginning of Section 1. We estimated the underlying survival functions for the probable Alzheimer’s disease group (389 participants, approx. 21% censoring), possible Alzheimer’s disease group (253 participants, approx. 24% censoring) and vascular dementia group (172 participants, approx. 19% censoring), separately, and computed bootstrapped 95% pointwise confidence intervals. The stacked estimator included the corrected NPMLE, Weibull, Log-Normal and Gamma estimators (See Additional file 3 for individual parametric estimates of survival). We did not include the Log-Logistic distribution in the stacking procedure as it admits only a decreasing hazard function, and is therefore not suitable as a model for survival with dementia. In Fig. 1, we plot the stacked estimates with 95% pointwise confidence limit curves in red along with the corrected NPMLE and the associated 95% pointwise confidence limit curves in black.

From Fig. 1, the stacked estimate generally captures the same shape as the NPMLE until approximately 60 months. The 60 month mark corresponds to the approximate follow-up time for subjects in the study and thus the non-parametric estimate does not capture the underlying survival function behaviour past this point. On the other hand, since the stacked estimator is defined as a linear combination of both the corrected NPMLE and parametric estimators with support unconstrained by the observed data, the resulting stacked estimate captures the survival function tail behaviour past 60 months. Additionally, from Fig. 1, we see that the bootstrapped confidence intervals based on the stacked estimator and the NPMLE are approximately of equal width in the case of the probable Alzheimer’s group. In the possible Alzheimer’s group, the widths based on the stacked estimator are visibly reduced, while the widths are strikingly reduced in the vascular dementia group. We note that in the possible Alzheimer’s disease group, decline in the first 60 months appears to be more rapid than in the other two groups. We speculate that the possible Alzheimer’s disease group included a variety of non-Alzheimer’s disease dementias, some of which are characterized by rapid decline.

In the probable and possible Alzheimer’s disease groups, the Weibull model received most of the weight, while in the vascular dementia group, the Gamma model was heavily favoured. This demonstrates the ability of the stacking model to shift its assignment of weight to a different model in the stack, a model (such as the Gamma) for example, that may not have been considered alone initially. For a listing of the individual submodel weights of the stacked models, see Table 5. The median survival estimated for all three dementia types was roughly 4.2 years when using a stacking model for the residual lifetimes. In comparison, the estimated median survival for the three dementia groups combined was roughly 4.5 years when using the full data that included the current lifetimes [3]. The latter (full) data, naturally, produced much narrower pointwise confidence intervals.

**Table 5:** Weights of stacked survival models applied to the three dementia type strata of the Canadian Study of Health and Aging

	Individual submodel type of stacked estimator			
CSHA Strata	NPMLE	Weibull	Log-Normal	Gamma
Probable Alzheimer’s Disease	1.282×10^−8^	0.9928	2.523×10^−7^	0.007240
Possible Alzheimer’s Disease	1.000×10^−8^	6.556×10^−7^	1.353×10^−7^	0.9999
Vascular Dementia	1.126×10^−8^	0.7179	2.022×10^−7^	0.2821

## Discussion

We originally hoped to improve the corrected NPMLE when estimating the survival function using only the observed residual lifetimes from a prevalent cohort study with follow-up. Our goal was to introduce parametric models into a stacking estimator, while retaining the corrected NPMLE, speculating that the parametric models would mitigate the two major shortcomings of the corrected NPMLE: (i) the wide pointwise confidence intervals that are often produced, and (ii) the failure of the corrected NPMLE to capture the tail behaviour of the survival function, particularly when follow-up is short. However, we found that when comparing the estimators using, essentially, their average DISSEs, the corrected NPMLE did not perform well either alone or as a member of the stack when follow-up was short.

The sample mean discrete integrated squared survival error takes into account both bias and variance. Nevertheless, our application suggests that even though confidence interval width is concerned only with variance, the stacked estimator produces narrower (sometimes considerably narrower) confidence intervals than those of the corrected NPMLE. A potential objection to the use of parametric models in the setting of this article, is their lack of robustness to model misspecification. By building the stack with several different parametric models, we believe that to a large extent, these fears can be allayed. It is comforting to see that in our example, the survival function produced by the stacking estimator is smooth, in that it does not have difficult-to-explain kinks.

An alternative approach for modelling the underlying survival function is through a parametric mixture model. Rather than fitting the individual parametric models separately for the residual lifetime density functions and then subsequently estimating the weights, one can define a mixture model likelihood function and then maximize the likelihood to estimate the failure time parameters and model weights simultaneously [19]. In contrast, it is possible to define a parametric mixture model of the submodel survival functions and then estimate the unknown parameters by maximizing the corresponding likelihood function. In both proposed approaches however, it remains an area of future research as to how to incorporate the non-parametric estimates into the mixture models and how these various procedures compare when predicting the underlying survival function. It is worth noting that multi-state models are often applied to survival (or event history) data. However, their use is somewhat limited under stationarity and it is hard to see how their introduction would enhance the proposed methods.

## Supplementary Information

**Additional file 1** Supp1-RFunctionsCode.R

**Additional file 2** Supp2-RSimulationsCode.R

**Additional file 3** Supplementary Figures.

## Data Availability

Dr. Christina Wolfson (christina.wolfson@mcgill.ca, Department of Epidemiology, Biostatistics and Occupational Health, McGill University) can be contacted concerning access to the data that support the findings of this study. The data are not publicly available. All simulation R code applied in manuscript available in Additional Files 1 and 2.

## Abbreviations

CSHA: Canadian study of health and aging
DISSE: Discrete integrated squared survival errors
NPMLE: Nonparametric maximum likelihood estimator
MLE: Maximum likelihood estimator
PDF: Probability density function
