# Supplementary material for: Stacked survival models for residual lifetime data
Source: BMC Med Res Methodol. 2022 Jan 7;22:10. doi: 10.1186/s12874-021-01496-3 (PMC8742399; doi:10.1186/s12874-021-01496-3)
Supplement: Supplementary file 3 — Additional file 3 Supplementary Figures. [file 12874_2021_1496_MOESM3_ESM.pdf]

Stacked Survival Models for Residual Lifetime Data

Supplementary Figures

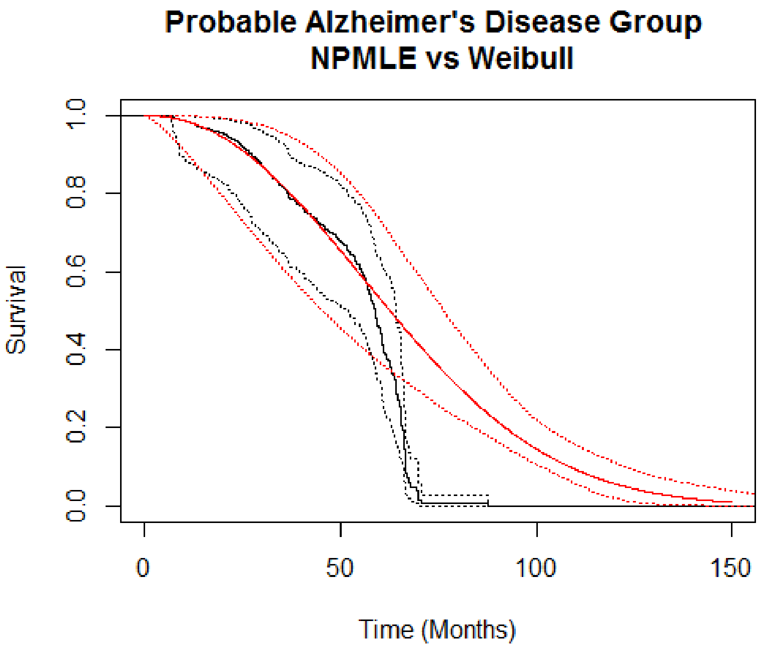

Figure S1: Estimated survival curves (solid lines) for the Probable Alzheimer's Disease Group using the NPMLE (black) or Weibull model (red) with corresponding 95% pointwise confidence intervals (dotted lines)

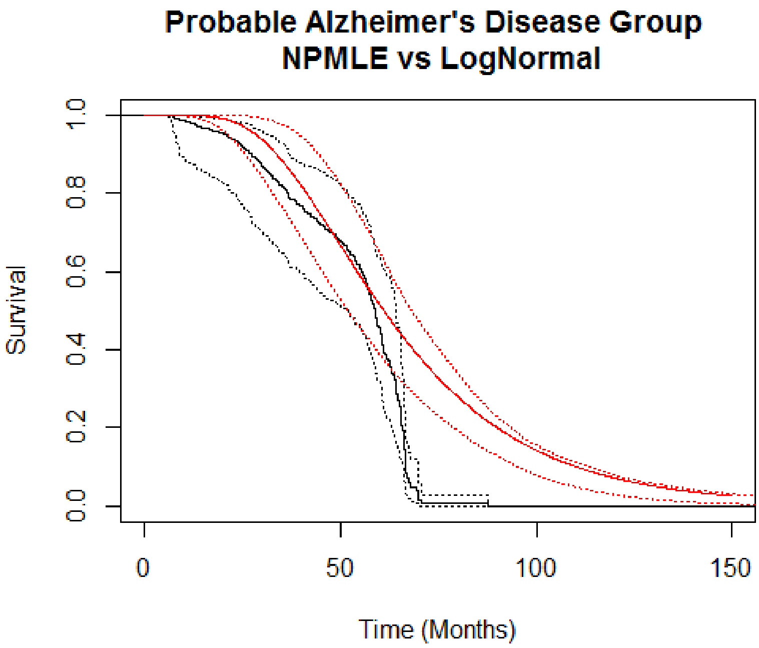

Figure S2: Estimated survival curves (solid lines) for the Probable Alzheimer's Disease Group using the NPMLE (black) or LogNormal model (red) with corresponding 95% pointwise confidence intervals (dotted lines)

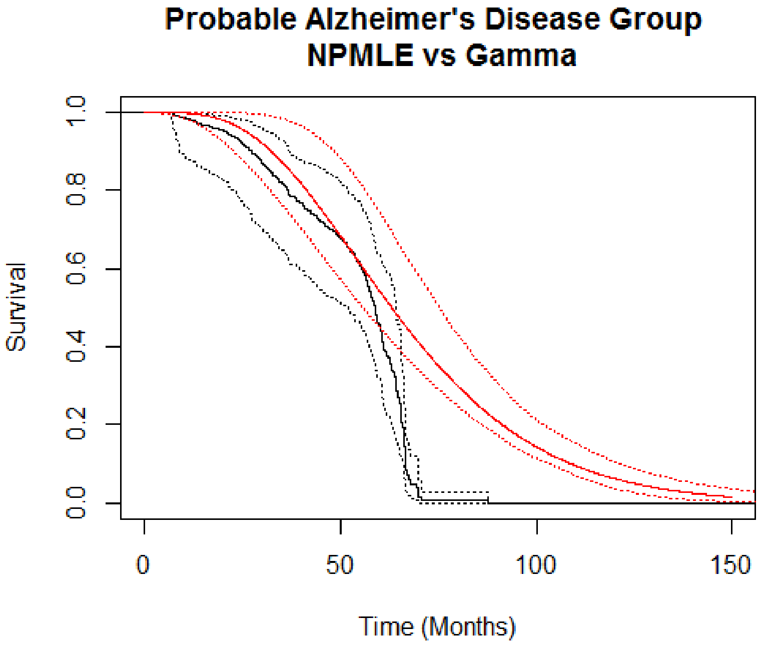

Figure S3: Estimated survival curves (solid lines) for the Probable Alzheimer's Disease Group using the NPMLE (black) or Gamma model (red) with corresponding 95% pointwise confidence intervals (dotted lines)

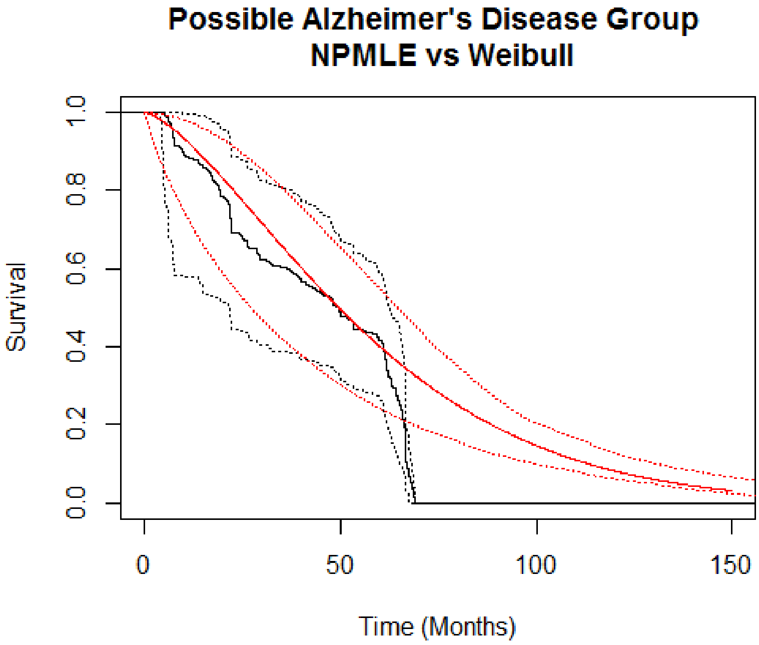

Figure S4: Estimated survival curves (solid lines) for the Possible Alzheimer's Disease Group using the NPMLE (black) or Weibull model (red) with corresponding 95% pointwise confidence intervals (dotted lines)

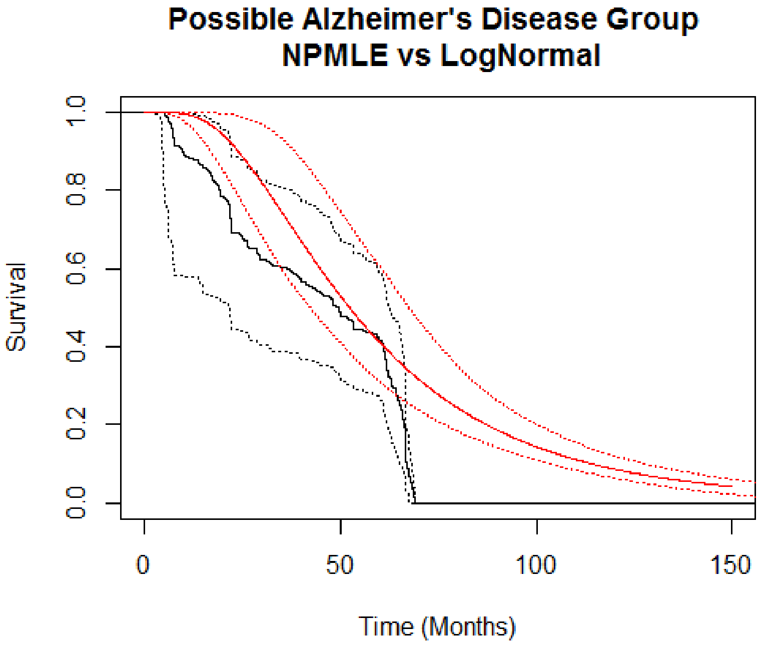

Figure S5: Estimated survival curves (solid lines) for the Possible Alzheimer's Disease Group using the NPMLE (black) or LogNormal model (red) with corresponding 95% pointwise confidence intervals (dotted lines)

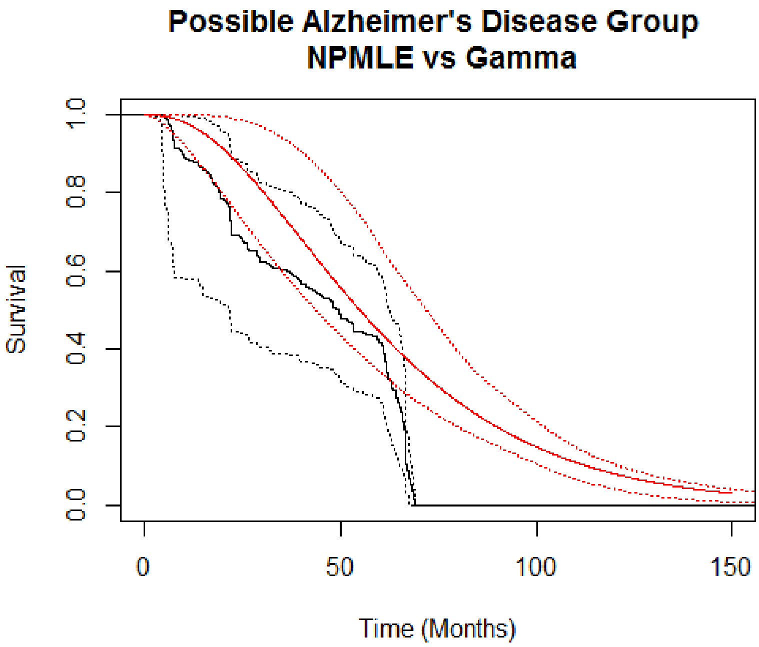

Figure S6: Estimated survival curves (solid lines) for the Possible Alzheimer's Disease Group using the NPMLE (black) or Gamma model (red) with corresponding 95% pointwise confidence intervals (dotted lines)

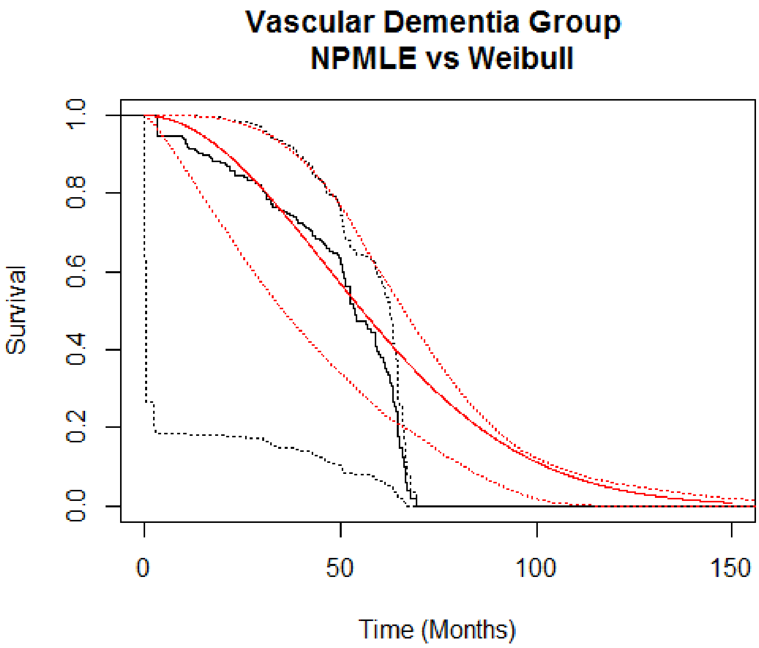

Figure S7: Estimated survival curves (solid lines) for the Vascular Dementia Group using the NPMLE (black) or Weibull model (red) with corresponding 95% pointwise confidence intervals (dotted lines)

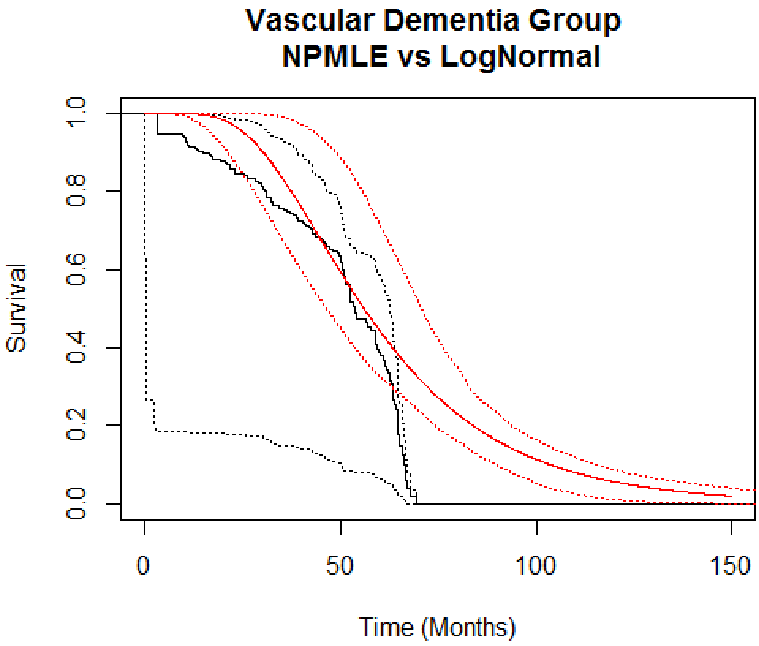

Figure S8: Estimated survival curves (solid lines) for the Vascular Dementia Group using the NPMLE (black) or LogNormal model (red) with corresponding 95% pointwise confidence intervals (dotted lines)

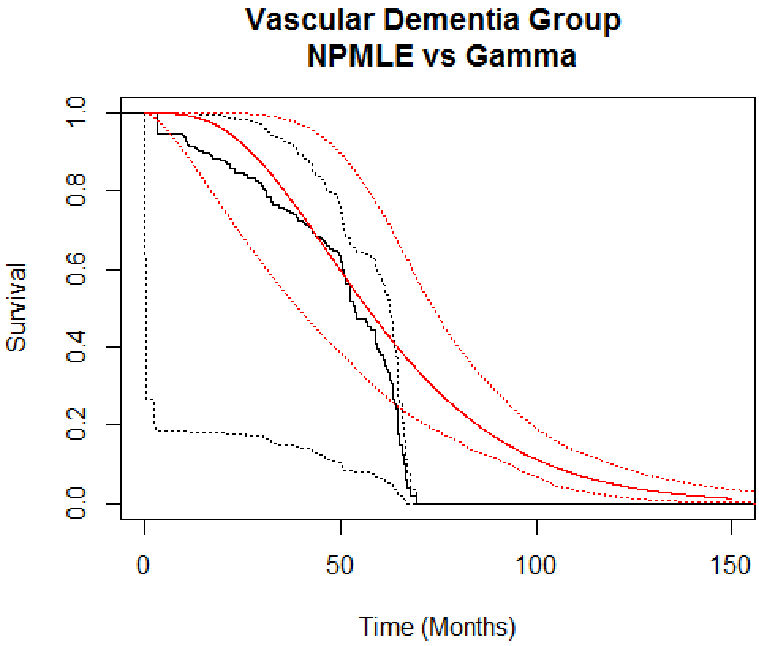

Figure S9: Estimated survival curves (solid lines) for the Vascular Dementia Group using the NPMLE (black) or Gamma model (red) with corresponding 95% pointwise confidence intervals (dotted lines)
